# Structures, Properties and Potential Applications of Corncob Residue Modified by Carboxymethylation

**DOI:** 10.3390/polym12030638

**Published:** 2020-03-11

**Authors:** Shishuai Gao, Yupeng Liu, Chunpeng Wang, Fuxiang Chu, Feng Xu, Daihui Zhang

**Affiliations:** 1 National Engineering Laboratory for Biomass Chemical Utilization; Key Laboratory of Chemical Engineering of Forest Products, National Forestry and Grassland Administration, Key Laboratory of Biomass Energy and Material, Institute of Chemical Industry of Forest Products, Chinese Academy of Forestry, Nanjing 210042, China; gaoshishuai1006@163.com (S.G.); liuyplhs@163.com (Y.L.); wangcpg@163.com (C.W.); 2 College of Materials Science and Technology, Beijing Forestry University, Beijing 100083, China; 3 Co-Innovation Center of Efficient Processing and Utilization of Forest Resources, Nanjing Forestry University, Nanjing 210037, China

**Keywords:** natural fibers, biocomposites, carboxymethylation, adhesive

## Abstract

In this study, corncob residue (CR) valorization was simply and efficiently realized via carboxymethylation, and its enhanced performance as fillers in urea-formaldehyde (UF) resin was investigated. The structures of corncob residue and carboxymethylated derivative were analyzed by nuclear magnetic resonance (NMR), Fourier-transform infrared spectroscopy (FTIR), and Raman techniques, respectively. The thermal stability, morphology, viscosity control, and adhesive strength were then investigated to evaluate its performance as fillers in UF resin composite. Similar to commercial flour, carboxymethylated CR could effectively disperse in UF resin. It also exhibited a better initial viscosity control between 30 and 50 °C. The adhesive test analysis showed that the shear strength of resin with carboxymethylated CR addition could reach 1.04 MPa, which was comparable to flour (0.99 MPa) and significantly higher than raw CR (0.45 MPa). Moreover, a low formaldehyde emission was observed.

## 1. Introduction

The utilization of natural fibers in composites has gained much popularity in recent years. Natural fiber reinforced composites display several advantages as compared to composites filled synthetic fibers such as potentially lower cost, lower density, and good specific strength and stiffness. Furthermore, the growing concern about environment problems and the potential shortage of fossil resources have promoted the interest in developing sustainable materials. Therefore, many scientists have investigated the possibilities to prepare composites with plant fibers such as flax, cotton, hemp, corncob and various grasses [1,2,3]. Currently, the agricultural and forestry residues have been considered as the rich and important natural fibers resources. Despite the development of regulations in China, most residues are burned without efficient utilization, causing serious environmental pollution. As one of the main grain crops, corn production was about 2.2 × 10^8^ t in 2017 in China. Therefore, about 3.5 × 10^7^–3.9 × 10^7^ t corncob could be obtained every year from the corn production. [4] Due to its richness in pentosans, corncob has been widely employed as a high-class quality and one of the most abundant feedstocks for xylitol production. [5] The process involves a simple separation of hemicellulose from corncob by hydrolysis. [6] Although xylitol production is economically feasible, the abundant corncob residue (CR) produced during the hydrolysis process is usually considered as solid wastes to directly be burned. This would inevitably lead to environmental issues and be harmful to the sustainable development. Currently, CR has been explored to produce hydrochar, carbon quantum, ethanol, biosorbent, and porous carbon in the literature [4,7,8,9,10]. However, these CR derived products are far from industrial applications due to their poor performance and high cost of production. Therefore, it is extremely attractive to develop an efficient approach for efficient utilization of the CR to a commercial level.

Urea-formaldehyde (UF) resin as an important polymeric resin has been widely and commercially used in various fields, including construction, agriculture, and for its environmental aspect. [11,12,13,14] The utilization of UF resin for the adhesion of wood and derived products has been attracting continuous interests, due to its good adhesive property, excellent stability, and low cost. [15] However, during the practical plywood production, flours, or starch are often added into UF resin precursors in order to improve the initial viscosity and pre-press strength of resin. [16,17] Despite their high performance, there was a concern due to the competitiveness with food resources. Therefore, extensive efforts have been devoted to developing non-food-based filler to improve the properties of UF resin composite, such as montmorillonite nanoparticle, nanoclay, nanosilica, and synthetic polymers [18]. However, the incompatibility with UF resin and poor viscosity control has greatly influenced their roles as a substitute to starch. Moreover, the synthetic polymers highly rely on petroleum resources. Therefore, it is highly desirable to find a renewable filler to replace starch without compromising its low cost and high performance.

Natural fibers, such as palm kernel meal, sago fibers, and lignocellulosic waste fibers, have been investigated as fillers to prepare UF resin composite with improved performance [19,20,21]. However, their addition percentage is relatively low and high loading would prevent the effective dispersion in the particleboard [22]. Fiber dispersion has been identified as a major factor influencing the properties of fiber composite, and high addition increases their tendency to agglomerate. Good fiber dispersion was able to promote good interfacial bonding and reduce voids, leading to an enhanced performance. Previously, Zhang et al. reported that water-soluble carboxymethyl cellulose could significantly improve the initial viscosity, interface compatibility, and bond strength of starch adhesives [23]. Obviously, the incorporation of carboxylic acid was beneficial to the dispersion of cellulose in adhesives due to the electrostatic repulsion interaction [24]. Although cellulose fiber could be further separated from CR to prepare cellulose derivatives, this process increased the cost, and was also time-consuming. The direct carboxymethylation of CR with no separation steps to prepare UF resins has never been reported. Its effect on the properties of UF resins remains unknown. Thus, we hypothesized that CR after direct carboxymethylation could be used as an effective filler to improve the properties of UF resins composite in terms of enhanced interfacial compatibility and mechanical properties, high filler loading, and satisfactory initial viscosity control.

In the present study, CR was simply and efficiently utilized as filler via carboxymethylation modification. The physicochemical properties of CR and carboxymethylated CR were analyzed by various techniques, such as ^13^C cross polarization magic-angle spinning nuclear magnetic resonance (CP/MASNMR), X-ray diffraction (XRD), Fourier-transform infrared spectroscopy (FTIR), Raman, etc. Its performance as fillers in UF resin composite was then investigated in terms of thermal stability, morphology, viscosity control and adhesive strength. To evaluate the performance of carboxymethylated CR (CMCR), commercial flour and carboxymethyl cellulose prepared from CR were used as controls. It was observed that carboxymethylated CR could effectively disperse in UF resins. It exhibited a better initial viscosity control in comparison with flour. The adhesive test analysis and formaldehyde emission were also evaluated to investigate the roles of carboxymethylated CR. Due to the simple, cheap, and efficient modification process, carboxymethylated CR might be industrially applied in the production of UF resin composite for wide applications.

## 2. Materials and Methods

### 2.1. Materials

Corncob residue, a by-product of corncob refining, was friendly provided by Jinan Shengquan Group Share Holding Co., Ltd. (Jinan, China). Sulfuric acid, sodium hydroxide, sodium hypochlorite, glacial acetic acid, sodium chloroacetate, and phosphoric acid were purchased from purchased from Sinopharm Chemical Reagent Co., Ltd. (Shanghai, China). Urea, melamine, formaldehyde, acetylacetone, and ammonium acetate were all AR grade regents and purchased from Nanjing Reagent Chemical Co., Ltd. (Nanjing, China).

### 2.2. Composition Analysis

Ash content was determined according to GB/T 742-2008. Two grams of CR were accurately weighed into a constant crucible. It was then charred with electric heating furnace. Finally, carbonized CR was transferred to the muffle furnace and burned for 4 h at 900 °C.

The ash content of CR was calculated by the following formula:

$$X=\frac{m_{2}-m_{1}}{m}\times 100\%$$

where *X* is the ash content of CR, *m*_1_ is the weight of the constant crucible, *m*_2_ is the weight of crucible and ash, and *m* is CR weight.

Acid-insoluble lignin was determined by a modified method according to GB 02677.8-1994. Briefly, 1 g of CR was weighed accurately into a 100-mL four-neck round bottom flask containing 15 mL 72% sulfuric acid, equipped with a condenser, a stirring rod, and a thermometer for 2.5 h at 18–20 °C. Subsequently, the concentration of sulfuric acid was diluted to 3% with distilled water. The suspended slurry was boiled for 4 h to remove cellulose, hemicellulose, and acid soluble lignin of CR. The final insoluble fraction was filtered and washed with deionized water until the filtrate was neutral. Finally, the residue was dried to constant weight in an oven at 105 °C. Acid soluble lignin was obtained by ultraviolet absorption method according to GB/T 10337-2008. The UV absorption intensity of the above diluted filtrate was measured at 205 nm. The content of acid soluble lignin was calculated by the Lambert–Beer law:

$$W\%=\frac{A}{110}\times D$$

where *X* is the absorbance value of test sample, 110 is the absorbance coefficient [L/(g·cm)], and *D* is the dilution factor of filtrate.

### 2.3. Cellulose Extraction and Carboxymethylation

CR was bleached with a classic processing technique as follows [25]: 200 g CR were added into 600 g of deionized water and stirred for 5 min, followed by the addition of excessive glacial acetic acid and sodium chlorite. The reaction was stirred for 1 h at 75 °C under pH 3–4. Subsequently, the fibers were filtered and washed to neutral with distilled water. The bleached fibers were hydrolyzed in 2% sulfuric acid solution at room temperature for 2 h to remove residual hemicellulose. Finally, the fibers were washed with excessive amount of water to neutral again and dried in an oven at 60 °C to constant weight. The obtained cellulose powders were used as a raw material for the subsequent carboxymethylation.

Carboxymethylation of CR and C was carried out following a typical method [26]. For the mercerization process, 100 g of the dried CR or C were impregnated into fivefold weight of isopropanol. Next, 100 mL of 50% concentration of NaOH was added dropwise into mixture at room temperature, accompanied by a gentle magnetic stirring. Then, the reaction was performed for 1 h. After the alkalization process was finished, 100 g of sodium monochloracetate dissolved in three-fold weight of isopropanol were added dropwise into the suspension at 50 °C for 1 h under stirring. Then, the suspension was heated to 70 °C for 1 h. The slurry was neutralized with 60% acetic acid and then filtrated. The residues were washed by ethanol/water (80/20 *v*/*v*) six times and pure methanol three times to remove undesirable byproducts. Finally, the products were oven-dried at 60 °C. The remaining fibers were labeled as carboxymethylated corncob residue (CMCR) and carboxymethylcellulose (CMC), respectively.

### 2.4. Determination of Degree of Substitution

The DS of CMCR and CMC was determined by a modified method according to GB1886. 232-2016. Briefly, 1 g of CMCR or CMC was carbonized with an electric stove. The residue was transferred into a muffle and burned for 30 min at 700 °C. The residual solids obtained as sodium oxide were dissolved into 100 mL deionized water. Then, the solution was acidified by 50 mL of 0.05 mol/L sulfuric acid standard solution. Finally, the acidified solutions were titrated up to neutral with a 0.1 mol/L sodium hydroxide standard solution.

The *DS* of CMCR and CMC was calculated by the following formulas:

$$c=\frac{V_{1}\times c_{1}-V_{2}\times c_{2}}{m}$$

$$DS=\frac{0.162c}{1-0.080c}$$

where *V*_1_ and *V*_2_ are the actual volumes of standard sulfuric acid solution and standard sodium hydroxide solution, respectively. *c*_1_ and *c*_2_ are the concentration of standard sulfuric acid solution and standard sodium hydroxide solution. The weights of CMCR or CMC are represented by *m*. The millimolar mass of a glucose unit in cellulose is 0.162 and that of sodium carboxymethyl is 0.080.

### 2.5. Characterization

CR and C were modified with phenyl isocyanate derivatives to improve their solubility in THF before GPC analysis. The derivative procedure was as follows: 100 mg of dry CR or C were placed in a 20 mL reaction bottle with 10 mL of dry pyridine and 2 mL of 4-chloroisocyanatobenzene. The mixture was heated to 80 °C for 40 h. The reaction was terminated with 2 mL methanol. The resulting clear and viscous solution was cooled and precipitated by adding the solution to 100 mL of methanol. The precipitate was collected by centrifugation for 8 min at 3800 rpm. The solid precipitate was further purified by dissolution in acetone, followed by precipitation in a water:methanol mixture (80:20). The derivatives were dried in a vacuum oven at 60 °C overnight. For the GPC measurements, the derivatives were dissolved in THF at a concentration of 5 mg/mL. The sample solutions were filtered through a 0.22 μm filter, prior to the GPC measurement. GPC was performed at room temperature using a Malvern Viscotek 3580 System (Malvern Instruments Limited, Malvern, UK) equipped with Viscotek GPC2502 RI detector and a GPC 1007 pump. The columns were T6000M (Malvern Instruments Limited, Malvern, UK), General Mixed Org 300 × 7.8 mm^2^ (CLM3009). HPLC grade tetrahydrofuran (THF) was used as an eluent, and the flow rate was 1 mL/min. The calibration curve was obtained with monodispersed polystyrene (PSt) as the standard. For CMCR and CMC, molecular weights was measured using a Malvern Viscotek 3000 System (Malvern Instruments Limited, Malvern, UK). General Mixed Org 300 × 7.8 mm^2^ (CLM3000). HPLC grade water was used as an eluent, and the flow rate was 1 mL/min. The calibration curve was obtained with monodispersed polyethylene glycol (PEG) as the standard.

The FTIR spectra of all samples were recorded using an FTIR instrument (Nicolet, USA IS10, Thermo Fisher Scientific InC., Waltham, MA, USA) equipped with an ATR accessory. All FTIR spectra were collected at a spectrum resolution of 4 cm^−1^, with 32 co-added scans over the range from 4000 to 650 cm^−1^. A background scan was acquired before scanning the samples. The spatial Raman mapping was performed using a Raman imaging microscope (Thermo Scientific DXR, Waltham, MA, USA). Raman maps (scan range = 50 μm × 50 μm) were collected using a spatial resolution of 5 μm. The collected spectra were processed using noise filtering and normalization techniques. The MCR method developed by ONNICxi software (Thermo Fisher Scientific InC., Waltham, MA, USA) was applied to mapping the distribution of COOH groups. ^13^C CP/MAS NMR experiments were performed on a Bruker AVANCE III 600 spectrometer (Bruker, Karlsruhe, Germany) at a resonance frequency of 150.9 MHz.^13^C CP/MAS NMR spectra were recorded using a 3.2 mm MAS probe and a spinning rate of 12 kHz. A contact time of 3 ms and a recycle delay of 3 s were used for the ^13^C CP/MAS measurement. The chemical shifts of ^13^C were externally referenced to TMS. Thermogravimetric analysis (TGA) was performed by NETZSCH TG 209 F1 thermogravimetric analyzer (Thermo Fisher Scientific InC., Waltham, MA, USA). The samples were heated from 40 to 800 °C at a rate of 10 °C/min under nitrogen atmosphere. A wide-angle X-ray scattering (PANalyticalX’Pert Pro MPD diffractometer, PANalytical B.V., Netherlands) was used to investigate the crystallization of raw materials and modified products. The milled and powdered samples were analyzed at ambient temperature using a Cu Kα X-ray source (40 kV, 40 mA) with a wavelength (λ) of 1.5405 Å, in the angular range from 10° to 60° 2θ by a step of 0.02 °/s. The index of the sample determination was carried out using a Jade 6.0 program (Bruker, Madison, WI, USA). The morphology of all samples, sputtered with gold with E-1010 ion-covered plane, was studied by a S-3400-SEM (Toshiba, Tokyo, Japan). Rheological measurements of UF resins with different fillers were performed on a HAAKE MARS III oscillatory rheometer (Thermo Fisher Scientific InC., Waltham, MA, USA) with parallel plate’s geometry. The plate diameter used was 20 mm, and the gap between the plates was 1 mm. The samples were placed between plates and sheared. Three replicates were used for each adhesive.

### 2.6. Synthesis of Melamine-Urea-Formaldehyde Resin

The molar ratio of F:(U+M) was 1.1:1 to synthesize UF resin. Firstly, formaldehyde (1200 g) was placed into a 2000-mL four-neck round bottom flask, equipped with a condenser, a stirring rod, and a thermometer, and then pH was adjusted up to 8.0 with 30% NaOH aqueous solution. Subsequently, 400 g of urea and 40 g melamine were weighted into the flask. Next, the mixture was heated to 90 °C under stir and refluxed for 30 min. The pH value of the reaction system was controlled in the range of 7.0–8.0. The second step was catalyzed by 20% phosphoric acid solution under thea pH range of 5.0–5.5. After the condensation, it was terminated by adjusting pH up to 7.0. Subsequently, 200 g of urea and 20 g melamine were added into the system to react for another 50 min. Finally, F/(U+M) mole ratios of UF resin was adjusted to 1.1 by adding the urea and melamine. Then, the resin was cooled to room temperature, and adjusted the pH to 8.0.

### 2.7. Preparation of Three-ply Plywood and Adhesion Test

Three-layer eucalyptus plywood panels were prepared as follows: the adhesive consisted of 100 parts resin, 1 part ammonium chloride as curing agent, and 20 parts fillers. The adhesive was artificially applied to the surface of the veneer with 180 g/m^2^. The plywood panels were pre-pressed at 0.8 MPa for 1 h under the ambient environment, and then hot-pressed at 1.2 MPa at 120–125 °C for 300 s. Finally, all panels were stored under ambient condition for 12 h before testing. For the adhesive test, three-ply plywood was cut into specimens (25 mm × 100 mm, gluing area of 25 mm × 25 mm). The bonding strength of the plywood was determined by electronic universal testing machine using the Chinese Standard GB/T 9846.3-2004. The testing speed was 5.0 mm/min. Fourteen plywood specimens of each plywood panels were submerged in water at 63 ± 2 °C for 3 h, and then cooled to 20 °C before the shear strength testing.

### 2.8. Formaldehyde Emission Test

The formaldehyde emission from the plywood was measured by a desiccator method as described in Standard GB/T 17657-2013. The emitted formaldehyde was absorbed by 300 mL of deionized water in a 240 mm diameter container. The formaldehyde concentration of the sample solution was determined by a Shimadzu Scientific Instrument (UV-1800, Shimadzu, Kyoto, Japan) using ammonium acetate and acetyl acetone solution method with colorimetric detection at 412 nm. The formaldehyde emission results were the average of three times tested in parallel.

## 3. Results and Discussion

**Scheme 1:**
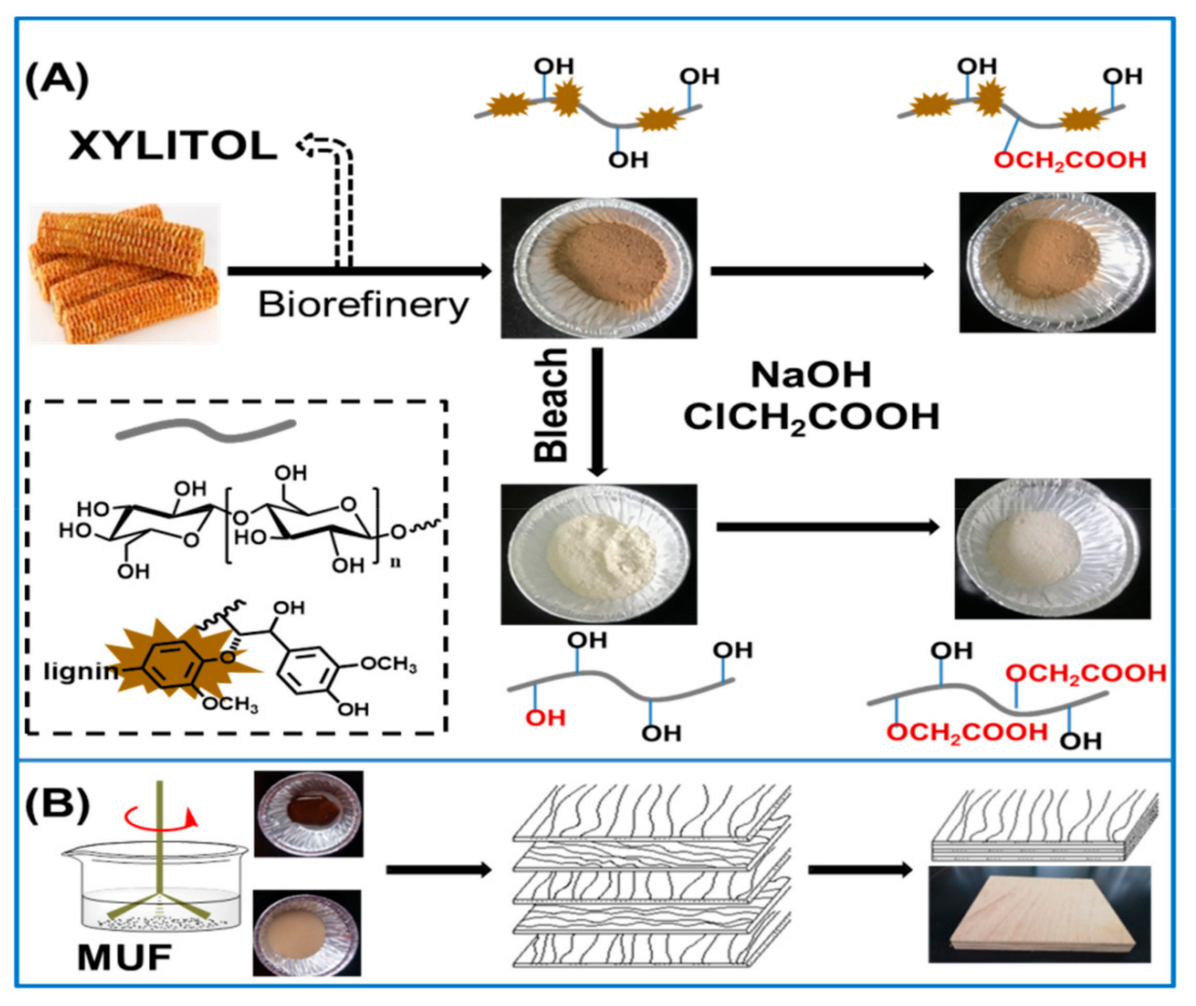
(**A**) Corncob waste valorizations via carboxymethylation; and (**B**) its application as filler in urea formaldehyde resin composite.

To improve the compatibility between corncob residue in UF resin composite, surface modification of substrates via carboxymethylation was used in this work. It was then used as a substitute for flour to effectively enhance performance of UF resin composite. As shown in Scheme 1, the corncob residue (CR) was directly carboxymethylated to introduce carboxylic acid groups (CMCR), which avoided the extra steps to remove residual lignin. In addition, commercial flour was chosen as a control since it has been widely and commercially used in the industry to improve the properties of UF resin. Moreover, carboxymethyl cellulose prepared from CR were also used as the control. Specifically, corncob residue was first bleached to remove lignin and hemicellulose, and then the obtained cellulose was carboxymethylated to carboxymethyl cellulose (CMC).

### 3.1. Carboxymethylation of Cellulose and Corncob Residue

Firstly, we analyzed the chemical compositions of corncob residues (Table S1). It was found that the cellulose content was 74.0% and the total lignin content was 22.2%. Most hemicellulose has been degraded after refining process and only 4.2% hemicellulose was remained. The presence of high content cellulose is beneficial to be utilized as a filler to improve the properties of UF resin composite. Furthermore, GPC analysis showed that the number average molecular weights of CR was 131,400 g/mol, as compared to 93,500 g/mol of bleached cellulose. Obviously, cellulose was partially degraded during the bleaching process. Then, both samples were subjected to carboxymethylation modification.

#### 3.1.1. Structural Characterization of Modified Substrates

**Figure 1:**
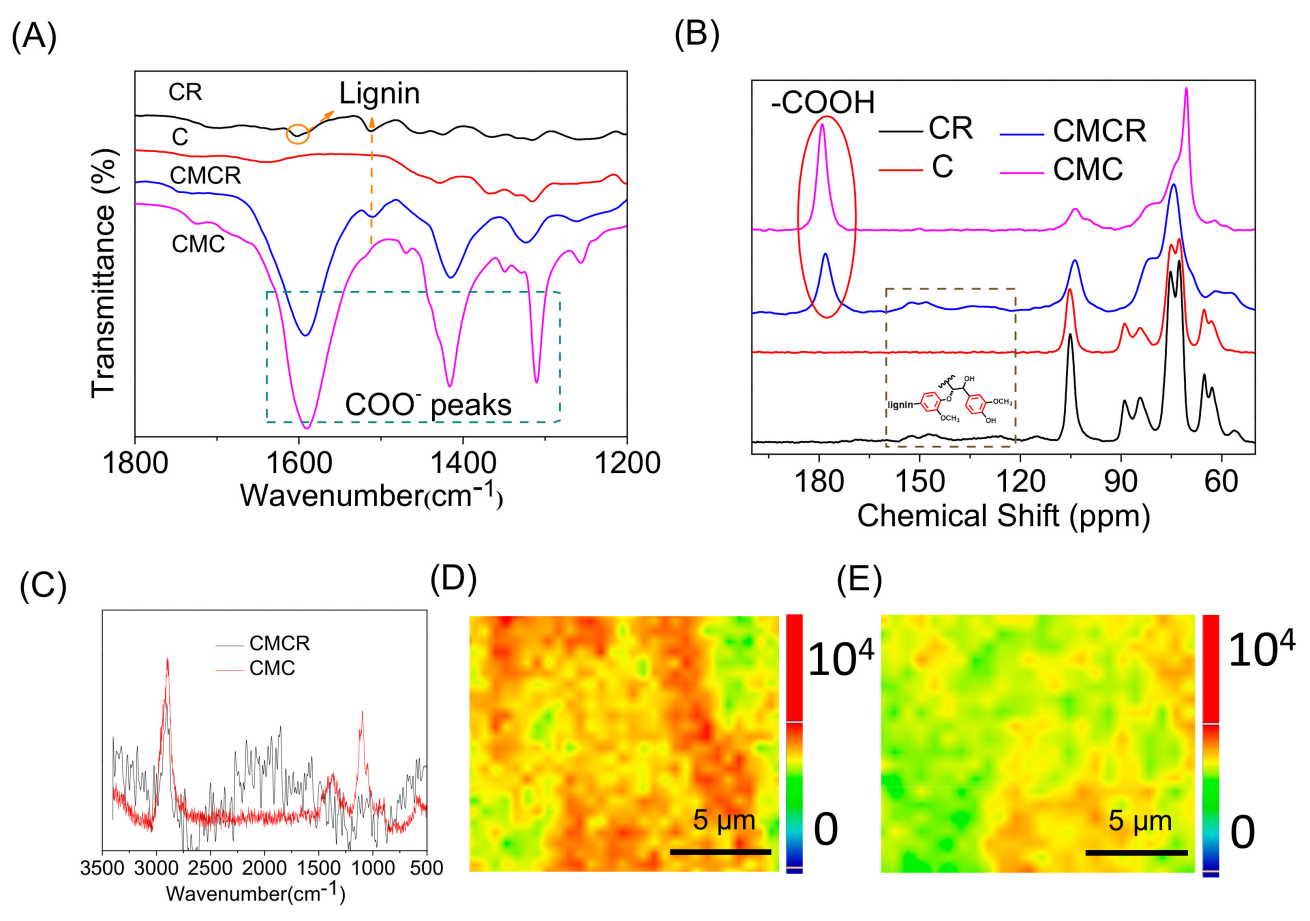
(**A**) FTIR spectra of CR (corncob residue), C (bleached cellulose), CMCR (carboxymethylated corncob residue), and CMC (carboxymethylated bleached cellulose) in the region 1800–1200 cm^−1^; (**B**)^13^C CPMAS NMR spectra of four samples; (**C**) Raman spectrum; and reconstructed Raman mapping of CMC (**D**) and CMCR (**E**) using –COOH as an indicator. The deep red spot corresponds to high intensity, whereas the blue spot indicates weak intensity.

FTIR was used to analyze the occurrence of carboxymethylation reaction on corncob residue and its bleached products. All samples showed typical characteristics peaks of cellulose backbone, such as a broad absorption band at 3356 cm^−1^ ascribed to the stretching frequency of OH groups and band at 2920 cm^−1^ due to the C–H stretching vibration (Figure S1). However, after modification, the intensity of –OH stretching signals was slightly decreased, revealing the occurrence of reaction between hydroxyl group and sodium monochloracetate. Furthermore, representative peaks for COO^–^ were observed in the region of 1600–1300 cm^−1^. There results indicate the successful modification of CR and C to their corresponding products (Figure 1A). In addition, ^13^C CPMAS NMR was performed to further affirm the incorporation of carboxylic acid groups. As shown in Figure 1B, the signal assigned to the carbon of carboxylic acid at 179.5 ppm was observed for CMC and CMCR. Moreover, by comparing CR with C, signals representing the benzene rings of lignin are also observable. We also further used Raman spectroscopy to visually demonstrate the spatial distribution of -COOH group in CMC and CMCR (Figure 1C–E). As compared to CMC, the Raman signal of CMCR was less smooth probably due to the complicated structures (Figure 1C). Figure 1D,E presents the 3D confocal Raman chemical imaging. The deep red color represented the highest content of –COOH. It should be noted that more scans of CMCR were needed to obtain a clear signal, even though CMC exhibited a large area of deep red as compared to CMCR in the preview area. This result clearly indicates the presence of residue lignin in the CR has negatively influenced the modification process, since most of hydroxyl groups were covered by lignin. In addition, it could be recognized that the distribution of carboxylic acid on the surface of substrates was not even, which might also be highly relevant to the distribution of lignin on the surface of fibers.

**Figure 2:**
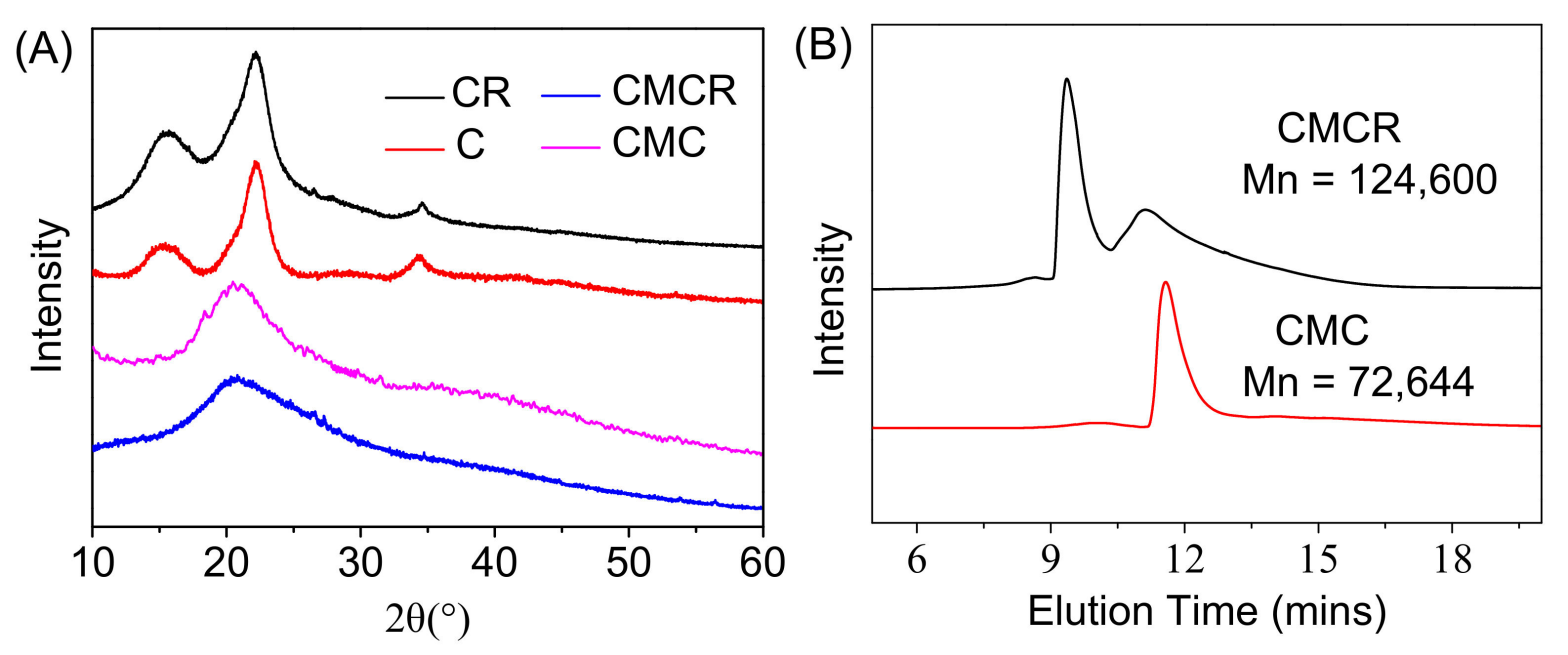
(**A**) XRD curves of CR, C, CMC and CMCR; and (**B**) GPC graphs of CMC and CMCR.

GPC analysis showed that the number average molecular weights of CMCR (*M*_n_ = 124,600) was higher than CMC (*M*_n_ = 72,644) (Figure 2A and Table S2). However, both values were significantly lower than the original samples, indicating the degradation of samples during the carboxymethylation process. XRD was used to evaluate the crystallinity variation of samples after different treatments (Figure 2B). Typical cellulose I characteristic peaks for CR and C at around 2θ = 15.7°, 22.1°, and 34.5° were observed, corresponding to (101), (002), and (400) lattice planes of cellulose I, respectively. As compared to CR, the crystalline diffraction peaks of C were more obvious. This was reasonable since the bleaching process was able to not only remove the lignin, but also destroy the amorphous region of cellulose [27]. After carboxymethylation, a broad peak at around 2θ = 20.8° was observed. This was consistent with other observation that the modification process could influence the intermolecular and intramolecular hydrogen bonding, leading to an amorphous structure [28,29,30]. Due to the enhanced dispersion of modified cellulose, it is beneficial to be used as a filler in UF resin without agglomeration. We also calculated the degree of substitution (DS), and it was 0.42 and 0.72 for CMCR and CMC, respectively. The DS of CMC was higher than that of CMCR. This result indicates that the presence of lignin negatively affected the modification process, which was consistent with Raman analysis.

#### 3.1.2. Thermal Stability of Modified Substrates

**Figure 3:**
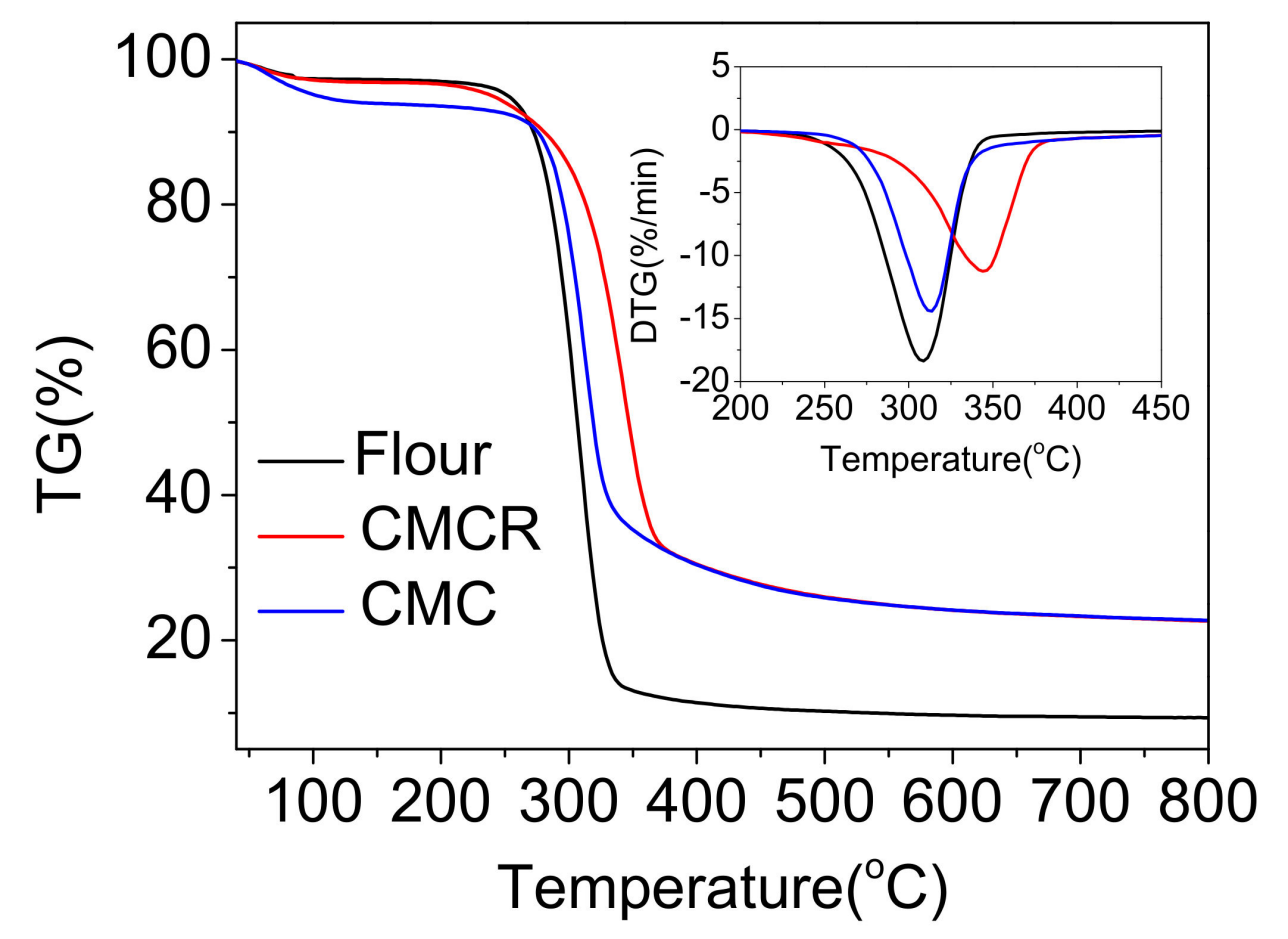
TGA analysis of flour, CMCR and CMC.

The thermogravimetric analysis (TGA) was utilized to evaluate the thermal stability. As shown in Figure 3 and Table S3, the thermal stability was varied depending on the compositions. Flour showed the highest *T*_5%_ (251.6 °C), while the highest *T*_max_ (344.4 °C) was observed for CMCR. Furthermore, it has been previously reported that *T*_5%_ and *T*_max_ of CR were around 200 and 350 °C, respectively [31]. Thus, the thermal stability of CMCR was comparable to the unmodified sample. As compared to CMC, CMCR exhibited an enhanced thermal stability probably due to the presence of lignin. The residues of CMCR and CMC degradation (22.8% and 22.6%) were higher than flour (9.3%).

#### 3.1.3. Morphology of Modified Substrates 

**Figure 4:**
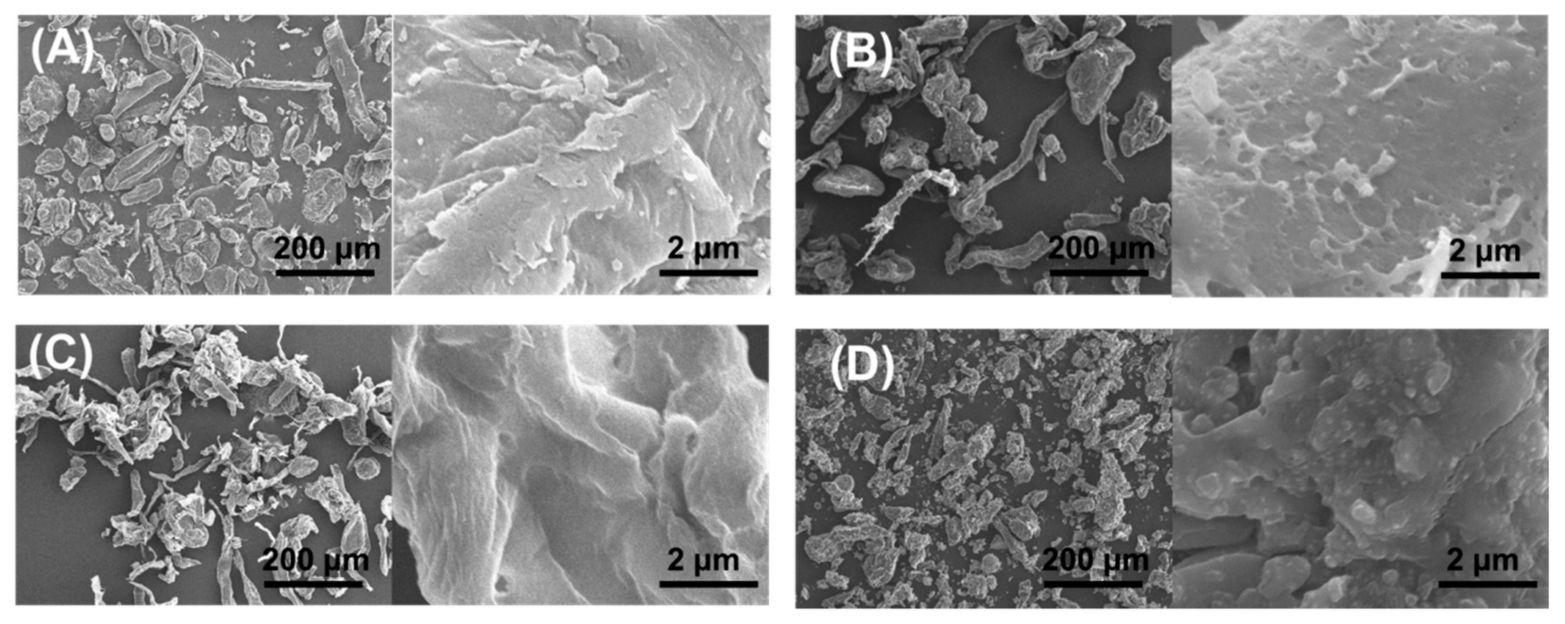
SEM images of: (**A**) CR; (**B**) CMCR; (**C**) C; and (**D**) CMC.

Visually, after bleaching, the color of corncob residue was changed from brownish to white due to the removal of lignin, hemicellulose, and other impurities. After carboxymethylation of CR, no significant variation in color was observed, indicating that the modification process failed to remove lignin and other residues. SEM was then used to observe the morphology variation of substrates in details. As shown in Figure 4, various small granule were observed on the surface of CR. After bleaching, the surface of C was relatively smoother as compared to CR, probably due to the removal lignin and others. After carboxymethylation of CR, the surface of CMCR particles also was smoother but the granules were still observable. As for CMC, the particles size was significantly decreased, and an uneven and rough surface was observed.

### 3.2. Enhanced Performance of Modified Substrates in UF Resin Composite

#### 3.2.1. Effect on the Viscosity and Viscoelastic Properties

After the successful preparation of carboxymethylated fillers, it was then added into UF resin composite to enhance the performance of resin. We firstly performed a strain sweep to observe the linear viscoelastic region (Figure S2). It showed that, for a deformation between 0.01% and 10.0%, the values of G’ practically remain constant for UF resin with CMCR addition, which did not depend on the value of the deformation, indicating a structural stability of resins. However, for the resin with flour addition, the linear viscoelastic region was 0.01–1%. Beyond 1.0%, changes occur in the resins structure (ruptured hydrogen bonds or entanglements), resulting in a decrease in the modulus. These results indicate that CMCR was able to improve the strain stability of resin composite. In addition, all rheological analyses were done on samples which were deformed by 1%.

**Figure 5:**
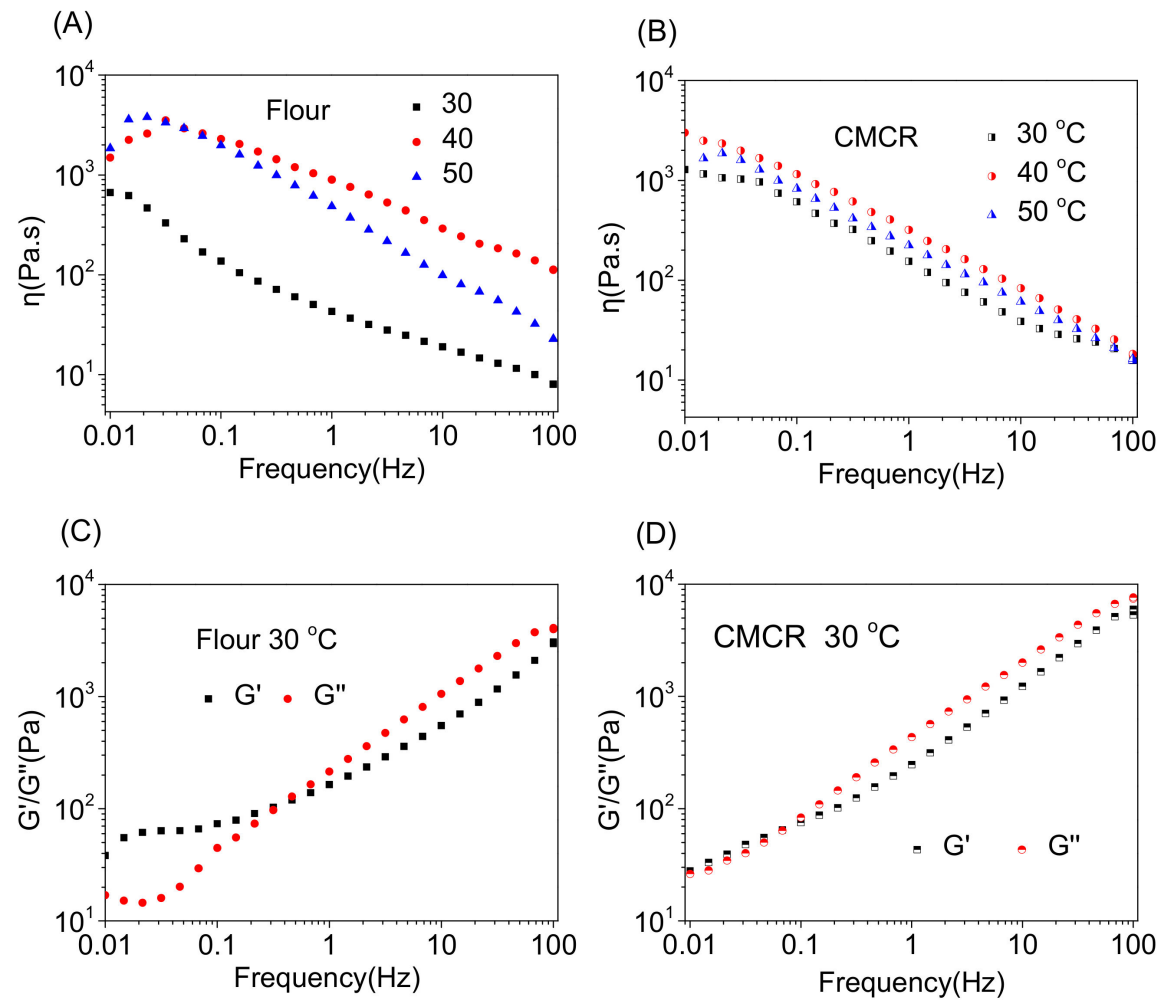
Viscosity-frequency curves of flour (**A**) and CMCR (**B**) at different temperatures; and viscoelasticity–frequency curves of (**C**) flour and (**D**) CMCR at 30 °C.

Industrially, to improve the production efficiency, UF resin composite containing fillers are usually applied to the surface of the wood veneer under a high shear. The ability of filler to provide a good thixotropy to the adhesive system is essential. Therefore, the blending system should be a typical pseudoplastic fluid. Its viscosity needs to be significantly reduced at a high shear, ensuring that the veneer is completely contacted and wetted by the adhesive. After shearing, the adhesive can quickly solidify, which prevents the adhesive from excessively penetrating into the pores of the veneer, resulting in a discontinuous layer of glue. In addition, the temperature varies greatly for different seasons, which can also influence the applications of UF resins. Therefore, the effect of CMCR addition on the viscosity under different temperatures was investigated, using flour as a comparison (Figure 5A,B and Figure S3). For both fillers, as the increase in the frequency, the viscosity was gradually decreased, which was beneficial to the practical processing. Furthermore, CMCR exhibited a better initial viscosity control due to its relatively high viscosity under different frequencies. In addition, the temperature effect on the viscosity for flour was more significant than CMCR. The highest viscosity of resins was observed at 40 °C, which was greater than 30 and 50 °C. The potential reason was attributed to the dual effects of temperature and gelation. The former contributed to the reduction of viscosity, while the latter increased the viscosity of the resins. Figure 5C,D shows the relationships of storage modulus (G’) and loss modulus (G) of UF resins as a function of frequency and fillers. The dynamic moduli increased with the frequency. At a low frequency zone, G’ was greater than G, indicating its solid-like behavior. This might be due to the formation of weak network due to the presence of filler as physical crosslinking points. As the frequency increased, there was an intersection point where G was larger than G’, indicating a solution state. However, when the temperature was increased to 50 °C, the G’ was always greater than G, indicating a weak gel. This might be due to the occurrence of gelation during the analysis. At high frequency zone, G’ of resin with CMCR was higher than the one with flour, indicating a better filler-reinforcing effect [24].

#### 3.2.2. Effect on the Curing Process

**Figure 6:**
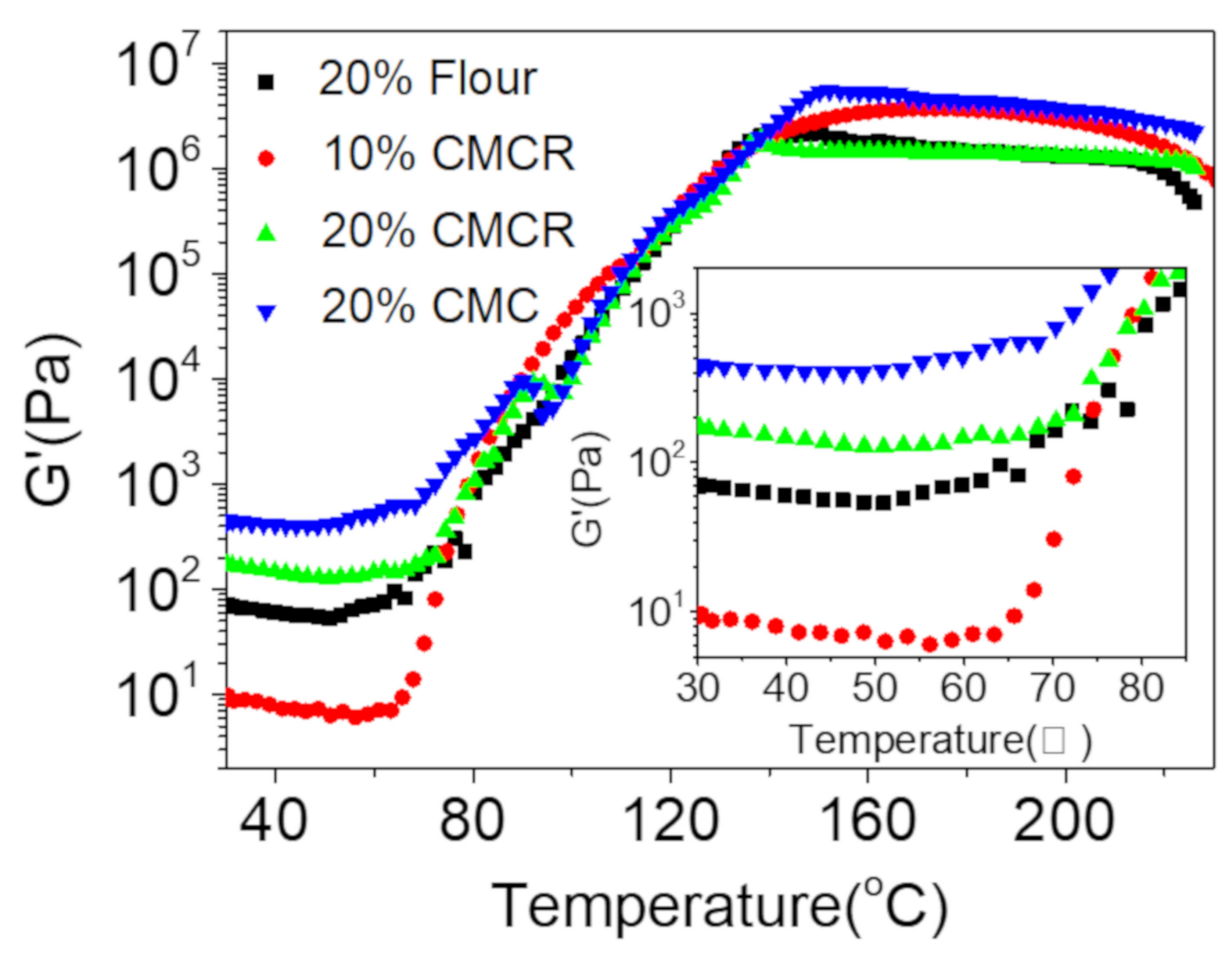
Variable temperature rheology analysis of UF resins with different fillers; 10% CMCR represents that the mass percentage of filler was 10%.

The variation of viscoelastic properties versus temperature could be used as an efficient indicator to monitor the curing process of UF resins composite. Thus, we utilized variable temperature rheology to understand the curing process. As for pure UF resin, the result is shown in Figure S5. As shown in Figure 6, four stages were observed, which was similar to pure UF resin. Firstly, as t the temperature increased from 35 °C to around 55 °C, the storage modulus was slightly decreased, indicating that its viscoelasticity was mainly controlled by temperature. However, in the second stage (55–145 °C), the modulus quickly increased mainly due to the occurrence of polymerization to UF resin. In addition, the evaporation of water was another reason to improve storage modulus. Subsequently, a relatively flat curve was observed in the range of 145–200 °C, probably indicating the end of polymerization reaction. A further increase in the temperature led to a decrease in modulus. This was due to the degradation of resin in air atmosphere. As compared to flour, both CMC and CMCR could enhance the initial modulus of UF resin solution. This was important since the enhanced modulus was beneficial to the practical operation and provide a high final mechanical strength. In addition, it was evident that CMCR concentration influenced the initial modulus. When 10% CMCR was added, there was an approximate ten times decrease in G’. This was reasonable since the addition of CMCR was able to increase the viscosity of UF solution.

#### 3.2.3. Effect on the Structural and Morphological Properties

**Figure 7:**
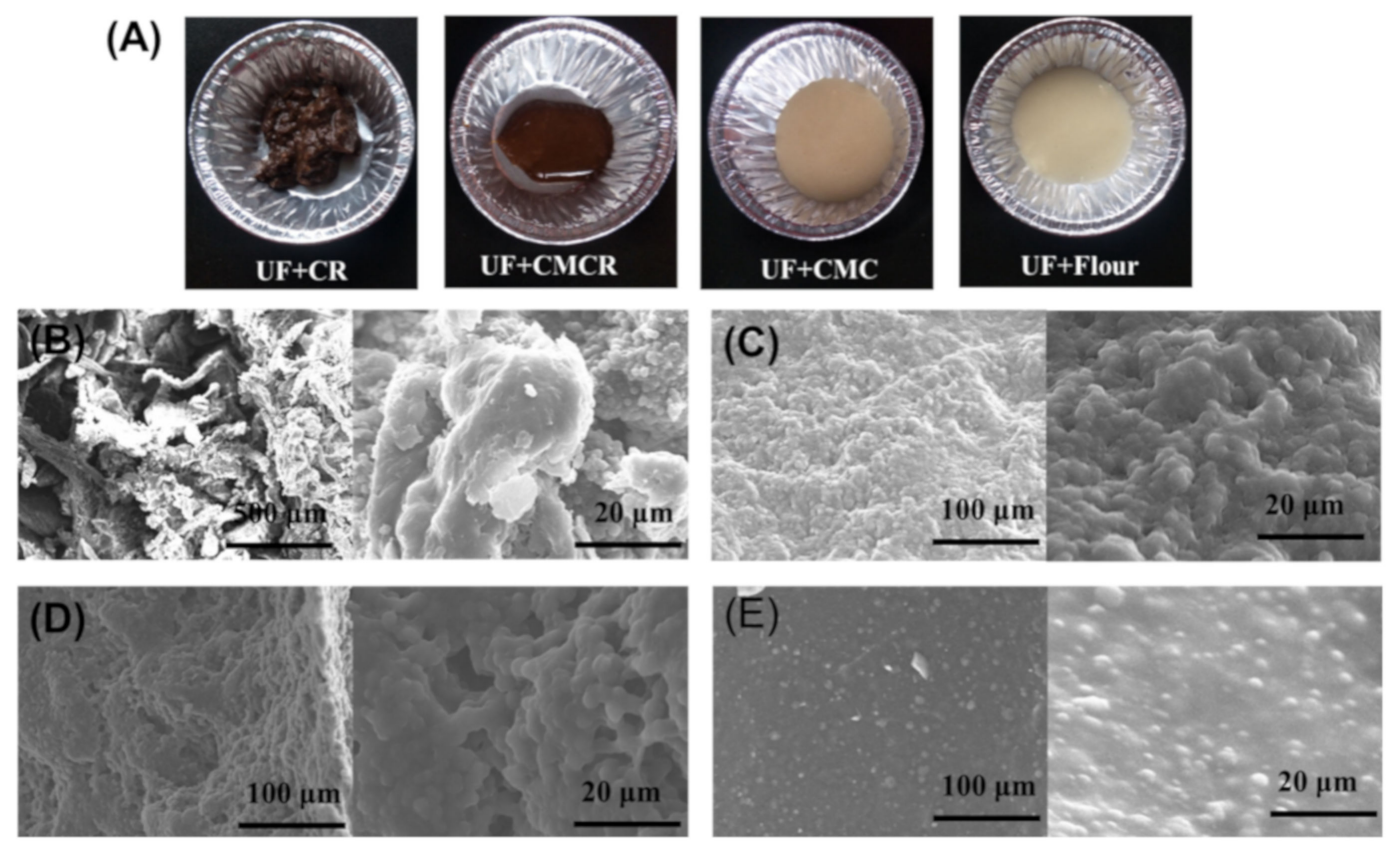
(**A**) Photographs of uncured UF resin with different fillers. Fracture SEM of the cured resins: (**B**) UF + CR; (**C**) UF + CMCR; (**D**) UF + CMC; and (**E**) UF + Flour.

We visually observed the difference after adding fillers into UF resin precursors solution (Figure 7A). For comparison, the pure UF resin is shown in Figure S6. An obvious aggregation of CR in UF resin was observed. For other fillers, due to the increased dispersion, a relatively homogenous solution was generated. In addition, UF with CMCR addition was brownish liquid due to the presence of lignin in the filler. Fracture SEM was used to observe the distribution of filler within UF resin. For CR, an obvious agglomeration phenomenon was observed. Moreover, the fiber could be easily recognized, and no resin dispersed on its surface, indicating the poor compatibility between CR and UF resin. This was consistent with the visual observation. After carboxymethylation, the compatibility was significantly improved. No agglomeration of cellulose fiber was observed. Oppositely, many spherical bumps were present. This result reveals that CMCR and CMC fibers could be completely covered by UF resin due to an enhanced interfacial compatibility. Moreover, this presence of large particles could contribute to the improvement in the adhesive strength since the they were able to penetrate into the pores of wood substrates and mechanically lock the adhesive and substrates. As for flour, a smoother surface was observed, together with evenly distributed small spherical particles. For pure resin, due to its brittleness, discontinuous sections were observed. The variation in the fracture surface was mainly due to the different structural features Although the carboxymethylation modification has influenced the crystalline structures of cellulose, the linear fiber structures largely remained, as compared to the amorphous structure of starch.

#### 3.2.4. Effect on the Thermal Stability 

**Figure 8:**
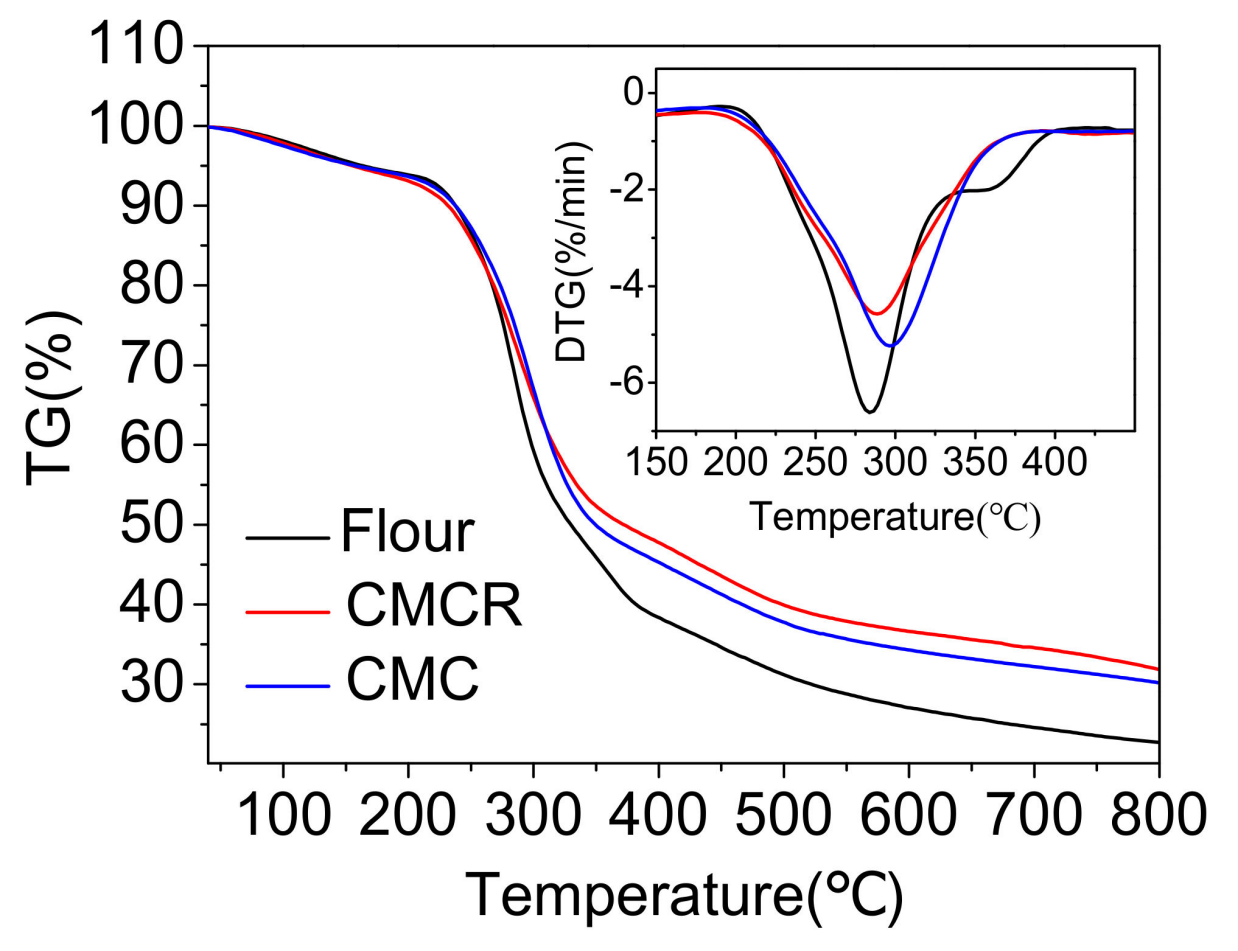
TGA analysis of cured resins filled with flour, CMCR and CMC.

Since the thermal stability was an important parameter for UF resin composite applications, the thermal stability of resins filled with flour, CMCR, and CMC was therefore investigated by TGA and derivative thermogravimetry (Figure 8). Similar degradation curves were observed when using different fillers. The initial slight weight loss was due to the evaporation of residual water from the samples. The T_5%_ of resins filled with flour, CMCR, and CMC was 163.1, 155.8, and 157.1 °C, respectively, which was lower than pure UF resin reported in a previous study (210 °C) [15]. The T_max_ of all resins could reach over 280 °C, which was similar to the pure UF resin. The result indicates that the thermal stability of resin filled with CMCR or CMC was comparable to the one with flour addition. In addition, the residues percentage of all samples was in the range of 22.7%–31.8%.

### 3.3. Adhesive Test

**Figure 9:**
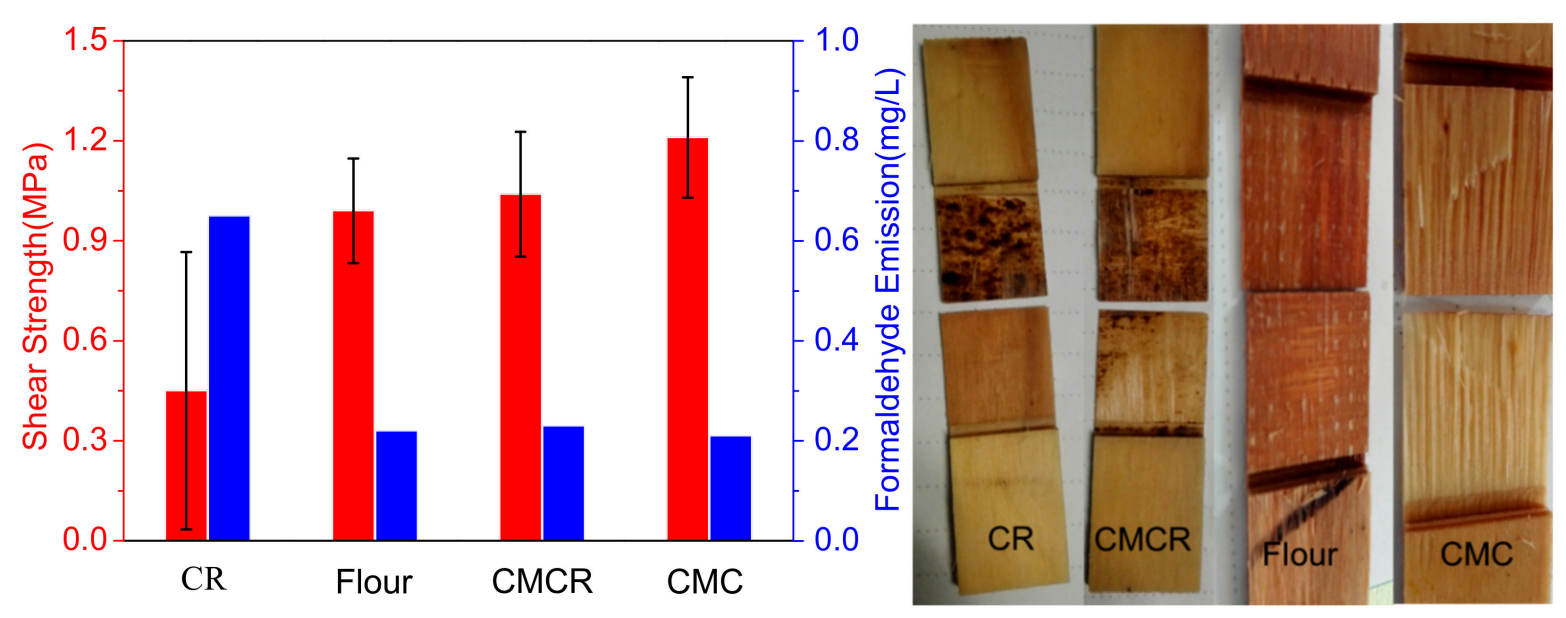
(**A**) Shear strength and formaldehyde emission of plywood; and (**B**) adhesive failure modes.

After curing, FTIR analysis confirmed the formation of UF resins due to the presence of typical characteristic peaks (Figure S4). The shear strength and formaldehyde emission of plywood were the most important parameters for UF resin composite as adhesive, which determined the quality and the application environment of the plywood. Thus, these properties were determined to investigate the effect of filler addition (Figure 9). The mass percentage of filler to UF resin was 20%. Without carboxymethylation, CR failed to uniformly disperse in UF resin due to the aggregation, and the shear strength was only 0.45 MPa (Figure 9A). Furthermore, the formaldehyde emission was highest, and it reached 0.65 mg/L. Since the pH of CR solution was around 7.2, the addition of CR might inhibit the condensation polymerization of UF resin and thus lead to the decreased mechanical properties and increased formaldehyde emission. When using CMCR as the filler, the shear strength was improved to 1.04 MPa, and the formaldehyde emission was only 0.20 mg/L. The presence of carboxyl acid groups could increase the electrostatic repulsion between cellulose chains, and thus enhance the dispersion of CMCR in UF resin. Moreover, the acid nature of carboxylic acid groups was also beneficial for the polymerization of UF resin (pH = 6.5). In addition, both parameters were comparable to widely used commercial flour (0.99 MPa and 0.22 mg/L). These results indicate that CMCR was a suitable substitute for flour. Although the shear strength was slightly lower than CMC (1.21 MPa), the easy preparation process enabled it to be more competitive for practical applications. Finally, we also tried commercial CMC as a control. Unfortunately, even when the mass percentage of commercial CMC was only 1%, a gel phenomenon was observed due to its high molecular weight and unexpected high pH value (7.6). As compared to pure UF resin reported in the previous study [15], resins with fillers showed similar shear strength and formaldehyde emission.

The failure mode was also investigated (Figure 9B). It could be found that there was an adhesive failure for UF resins with CR addition. Moreover, a serious aggregation of CR particles was present. This result was consistent with the mechanical properties and SEM analysis. Oppositely, an adhesive failure was observed for CMCR besides substrate failure, indicating its enhanced shear strength. Moreover, the distribution of CMCR in UF resin was obviously even more, further demonstrating that the modification process contributed to the effective dispersion of filler in the adhesive. For CMC, a main substrate failure occurred, explaining the high shear strength, while the failure mode for flour was adhesive failure.

## 4. Conclusions

Carboxymethylation of corncob residues (CR) was successfully performed and demonstrated its enhanced performance as filler in the application of UF resin composite. The modification process altered the crystalline structures of CR and caused the decrease in the molecular weights. While the presence of lignin influenced the degree of substitution, the carboxymethylated CR could still evenly disperse in UF resin due to the electrostatic repulsion. As a result, UF resin with carboxymethylated CR addition exhibited a better initial viscosity control between 30 and 50 °C, as compared to flour. Moreover, the adhesive test analysis showed that the shear strength of carboxymethylated CR could reach 1.04 MPa, which was comparable to flour (0.99 MPa) and significantly higher than CR (0.45 MPa). A low formaldehyde emission was also observed. These results indicate that carboxymethylation modification was an effective yet simple way to convert agricultural and forestry wastes to useful fillers for composite production.

## Supplementary Materials

The following are available online at https://www.mdpi.com/2073-4360/12/3/638/s1, Figure S1: FTIR spectra of CR, CMCR, C, and CMC in the 4000–2600 cm^−1^ region, Figure S2: Strain sweep of UF resins with flour and CMCR as fillers, Figure S3: Relationships between G’ and G’’ of UF resins with flour or CMCR as fillers and frequency sweep at different temperatures, Figure S4: FTIR spectra of cured UF resins with flour, CR, C, CMCR, or CMC as fillers, Figure S5: Variable temperature rheology analysis of pure UF resin, Figure S6: Fracture SEM of the pure UF resin, Table S1: Chemical compositions of corncob residues, Table S2: GPC analysis of CR, C, CMCR, and CMC, Table S3: Summary of TGA and DTG analysis, Table S4: Physical-chemical properties and plywood properties of UF resin with different fillers.
