# Supplementary material for: Structures, Properties and Potential Applications of Corncob Residue Modified by Carboxymethylation
Source: Polymers (Basel). 2020 Mar 11;12(3):638. doi: 10.3390/polym12030638 (PMC7183323; doi:10.3390/polym12030638)
Supplement: Supplementary file 1 [file polymers-12-00638-s001.pdf]

Summary of the Figures and Tables

Figures

Figure S1 FTIR spectra of CR, CMCR, C and CMC in 4000-2600 cm-1 region.

Figure S2 Strain sweep of UF resins with flour and CMCR as fillers.

Figure S3 Relationships between G' and G'' of UF resins with flour or CMCR as fillers and frequency sweep at different temperature.

Figure S4 FTIRspectra of cured UF resins with flour, CR, C, CMCR or CMC as fillers

Figure S5 Variable temperature rheology analysis of pure UF resin.

Figure S6 Fracture SEM of the pure UF resin.

Tables

Table S1 Chemical compositions of corncob residues.

Table S2 GPC analysis ofCR, C, CMCR and CMC.

Table S3 Summary of TGA and DTG analysis.

Table S4 Physical-chemical properties and plywood properties of UF resin with different fillers.

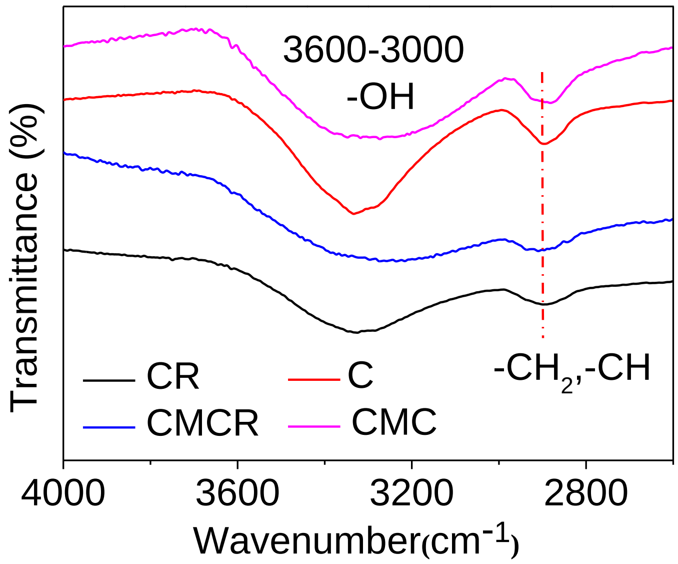

Figure S1 FTIR spectra of CR, CMCR, C and CMC in 4000-2600 cm^{-1} region.

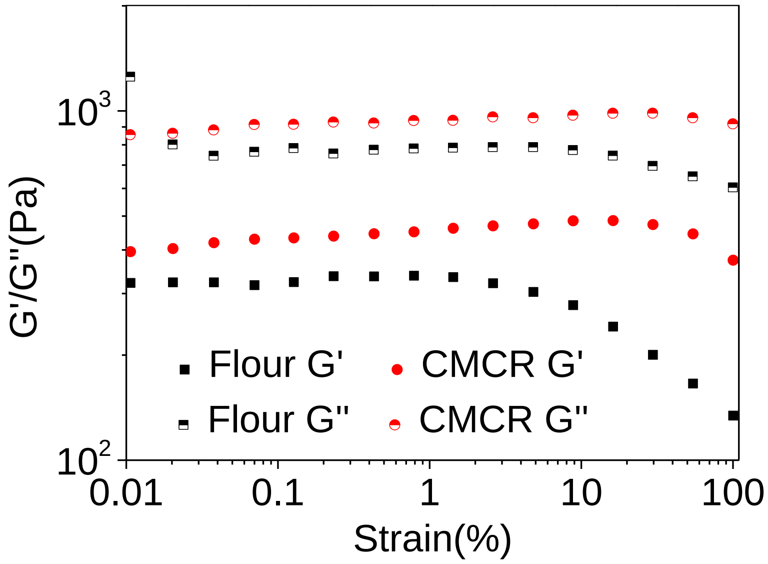

Figure S2 Strain sweep of UF resins with flour and CMCR as fillers.

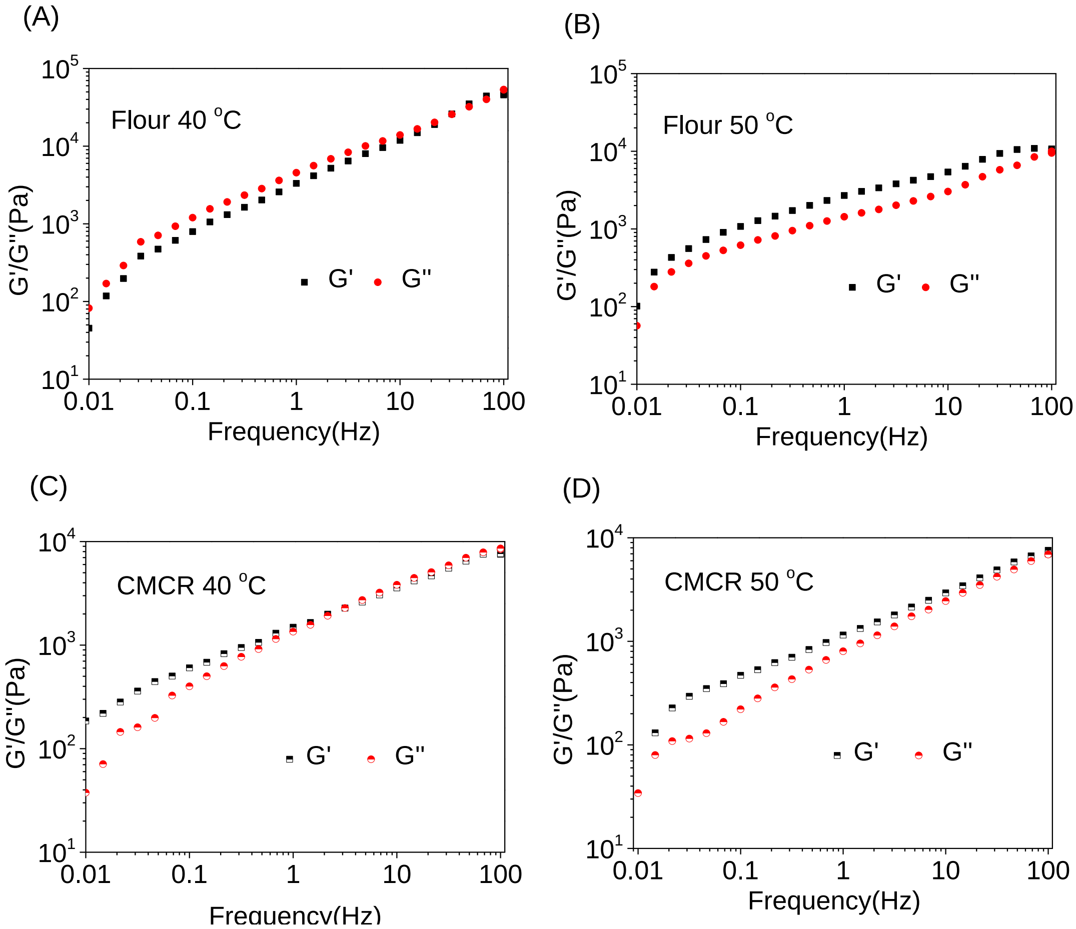

Figure S3 Relationships between G' and G'' of UF resins with flour or CMCR as fillers and frequency sweep at different temperature.

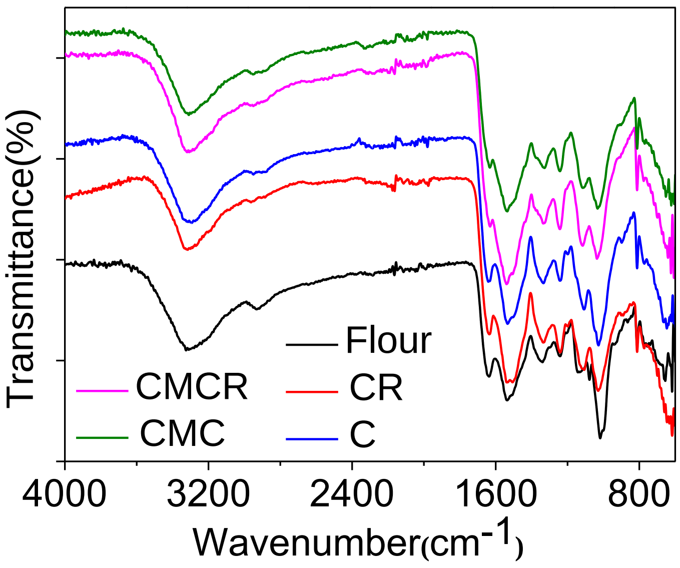

Figure S4 FTIR spectra of cured UF resins with flour, CR, C, CMCR or CMC as fillers.

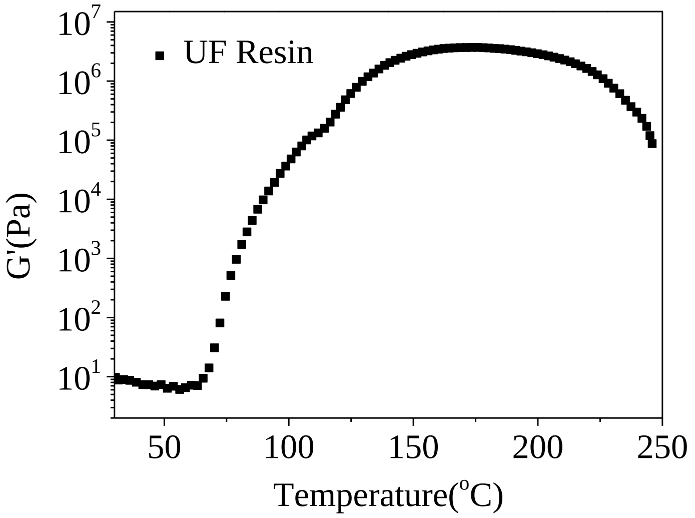

Figure S5 Variable temperature rheology analysis of pure UF resin.

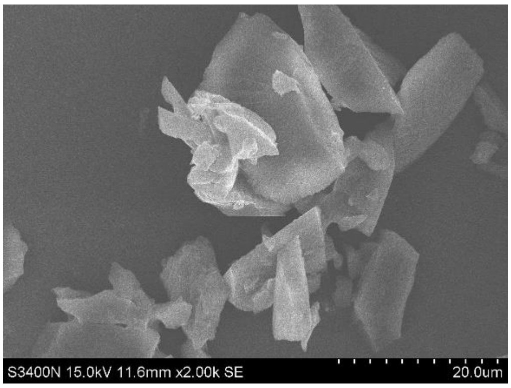

Figure S6 Fracture SEM of the pure UF resin.

Table S1 The chemical compositions of corncob residues.

Cellulose content/%	Acid insoluble lignin content/%	Acid soluble lignin content/%	Hemicellulose content/%	Ash content/%
74.04	22.06	0.11	4.21	2.90

Table S2 GPC analysis of CR, C, CMCR and CMC.

Sample	CR	C	CMCR	CMC
M_n	131,400	93,500	124,600	72,644
PDI	19.1	5.8	15.4	6.4
DS	/	/	0.42	0.72

Table S3 Summary of TGA and DTG analysis.

Sample	$T_{5\%}$(℃)	T_{max}(℃)	DTG_{max} (%/min)	R (%)
Flour	251.6	313.2	18.4	9.3
CMCR	238.9	344.4	11.2	22.8
CMC	103.2	316.9	14.6	22.6
MUF-Flour	163.1	283.7	7.0	22.7
MUF-CMCR	155.8	288.5	4.6	31.8
MUF-CMC	157.1	296.8	5.2	30.2

Table S4 Physical-chemical properties and plywood properties of UF resin with different fillers.

Species	Mass percentage (%)	pH value	η (mPa.s)	Shear strength (MPa)	Formaldehyde emission (mg/L)
Flour	20	6.7	4766	0.99	0.22
CR	20	7.2	46420	0.45	0.65
CMCR	20	6.5	8679	1.09	0.20
CMC	20	6.6	9346	1.21	0.21
Commercial CMC	1	7.6	gelling	/	/
